# Effects of Rest Position on Morphology of the Vastus Lateralis and Its Relationship with Lower-Body Strength and Power

**DOI:** 10.3390/jfmk4030064

**Published:** 2019-09-03

**Authors:** Alyssa N. Varanoske, Nicholas A. Coker, Bri-Ana D.I. Johnson, Tal Belity, Gerald T. Mangine, Jeffrey R. Stout, David H. Fukuda, Adam J. Wells

**Affiliations:** 1 Institute of Exercise Physiology and Rehabilitation Science, Division of Kinesiology, University of Central Florida, 12494 University Blvd., Orlando, FL 32816, USA; 2 Department of Exercise Science and Sport Management, Kennesaw State University, Kennesaw, GA 30144, USA

**Keywords:** cross-sectional area, muscle thickness, echo intensity, pennation angle, posture, strength and power, muscle morphology, subcutaneous adipose tissue thickness, supine, echogenicity

## Abstract

Ultrasonography of the lower body typically encompasses supine rest due to fluid shifts affecting tissue size and composition. However, vastus lateralis (VL) examination is completed in the lateral recumbent position, and this positional change may influence morphology and its ability to predict function. This study aimed to examine the effect of position on VL morphology and its relationship with lower-body performance. Cross-sectional area (CSA), muscle thickness (MT), pennation angle (PA), echo intensity (UnCorEI), subcutaneous adipose tissue thickness (SFT), and echo intensity corrected for SFT (CorEI) were assessed in 31 resistance-trained males (23.0 ± 2.1 yrs; 1.79 ± 0.08 m; 87.4 ± 11.7 kg) immediately after transitioning from standing to supine (IP), after 15 min of standing (ST), and after 15 min of rest in three recumbent positions: supine (SUP), dominant lateral recumbent (DLR), non-dominant lateral recumbent (NDLR). Participants also completed unilateral vertical jumps, isometric/isokinetic tests, and a one-repetition maximum leg press. CSA, MT, PA, and SFT were greater in ST compared to NDLR, DLR, and SUP (*p* < 0.05). CSA, UnCorEI, and CorEI were different between recumbent positions; however no differences were observed for MT, PA, and SFT. Different magnitudes of relationships were observed between muscle morphological characteristics measured after rest in different positions and performance variables. Muscle morphology in IP generally appears to be the best predictor of performance for most variables, although utilizing the NDLR and DLR positions may provide comparable results, whereas morphology measured in ST and SUP provide weaker relationships with physical performance. IP also requires less time and fewer requirements on the technician and subject, thus researchers should consider this positioning for VL examination.

## 1. Introduction

The assessment of muscle morphology in vivo has been used to evaluate muscle function in response to various exercise and nutritional interventions, as well as in disease and other clinical conditions. Previously, the use of computerized tomography (CT), magnetic resonance imaging (MRI), and dual-energy x-ray absorptiometry (DEXA) have been considered the gold standards in the assessment of muscle size and composition. However, ultrasonography has gained significant attention due to its ability to provide valid and reliable measures of both muscle size and fiber orientation [1,2,3,4,5,6,7]. Although less effective than radiology at imaging body cavities and bone, ultrasound devices are portable, versatile, and do not produce ionizing radiation [8,9]. Thus, ultrasonography represents a robust, non-invasive method of skeletal muscle imaging. 

Ultrasonography of the lower-body is typically completed while the subject is recumbent on an examination table; however, the transition from an upright to recumbent position has been shown to induce rapid fluctuations in blood flow and resulting tissue volume [10,11,12,13,14,15]. Specifically, a redistribution of blood out of the lower extremities and a decrease in hydrostatic pressure of the lower body result in a net absorption of fluid from the interstitial fluid into the capillaries, decreasing tissue volume [12,16,17,18,19,20,21]. Research has demonstrated that changes in body position result in alterations in muscle morphology of the lower body [10,11,14,22,23,24,25]. Due to this, ultrasonography is typically performed after a 10–15 min period of rest in the supine position to allow for gravitational fluid shifts to occur [1,26,27,28,29,30,31,32,33,34]. Nevertheless, Wagle and colleagues [15] recently observed stronger relationships between standing measures of muscle architecture and lower body strength and power when compared to measures taken while recumbent, which was attributed to discrepancies between the position during examination and the position in which many physical performance measures are evaluated. As muscle morphological characteristics obtained via ultrasonography appear to differ depending on whether they are obtained while standing or while recumbent [14,15,25], the ability of these characteristics to predict muscle function during upright activities may be compromised if ultrasound images are captured in the recumbent position. Nevertheless, ultrasonography in the standing position places an additional level of difficulty on the subject as well as the technician. The ability to compare measures of muscle morphology obtained from standing ultrasound scans to CT, MRI, and DEXA is also diminished because the latter of these techniques require participants to remain in the recumbent position during examination. Furthermore, ultrasound images captured while in the recumbent position may be altered by the rest position prior to assessment, affecting the ability of these images to predict muscle function. The vastus lateralis (VL) is a muscle that is commonly examined during the evaluation of lower body strength and power. Previous studies indicate that subjects are instructed to lay in the supine position for fluid shifts to occur and then flip over onto their lateral side for assessment [15,26,27,28,29,30,31,32,33,34]. However, changes in hydrostatic pressure and blood distribution may also be induced with changes in recumbent positions [35,36], and a change in position (from rest in a supine position to examination in a lateral recumbent position) may alter muscle morphological characteristics, which may not reflect true changes in muscle function.

Measurements obtained during ultrasonography may be further influenced by compression of body tissues as a result of changes in body position. In the examination of bilateral asymmetries in muscle size and architecture via ultrasonography, previous investigations do not report a return to the supine position for the same duration prior to examination of the opposing muscle [28,30]. Thus, the leg that was previously compressed under the weight of the body in the lateral recumbent position is then examined without the potential for additional fluid shifts to occur. Compression of a tissue increases the interstitial hydrostatic pressure, which reduces filtration of fluid out of the capillaries, therefore minimizing changes in muscle size [37]. It remains unknown whether compression of a limb under the weight of the body would affect muscle morphological characteristics. 

Consequently, it is possible that changes between recumbent positions affect muscle morphological characteristics of the lower body. Further, if differences in muscle morphology exist after rapid changes in body position, this may affect the relationship between these characteristics and muscle function. Therefore, the purpose of this study was to examine the effect of rest position on ultrasound-derived morphological characteristics of the VL and to determine whether the rest position that is used prior to ultrasound assessment affects the relationships between muscle morphological characteristics of the VL and lower-body force and power production.

## 2. Materials and Methods

### 2.1. Experimental Design

**Figure 1:**
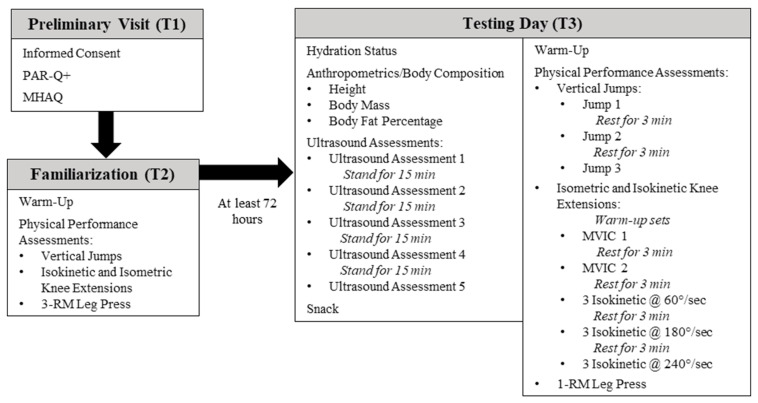
Timeline of study procedures. PAR-Q+: Physical activity readiness questionnaire; MHAQ: Medical history and activity questionnaire; RM: Repetition maximum; MVIC: Maximal voluntary isometric contraction.

Participants reported to the Human Performance Laboratory at the University of Central Florida on three separate occasions for this cross-over, correlational, prospective investigation. During visit one (T1), informed consent was obtained from all individual participants included in the study, and a medical history and activity questionnaire (MHAQ) and a physical activity readiness questionnaire (PAR-Q+) were completed to establish eligibility. During visit 2 (T2), participants underwent a familiarization session with all physical performance assessments to minimize any learning effect of the assessments on outcome variables. At least 72 h after T2, participants visited the laboratory for their final visit (T3), which consisted of hydration status assessment, anthropometric testing, body composition assessment, ultrasound assessments, and physical performance testing. A depiction of all visits to the laboratory and associated assessments is presented in Figure 1. This investigation was approved by the University of Central Florida Institutional Review Board for human subjects (approval number: BIO-18-14303; 21 September 2018), and all procedures were in accordance with the ethical standards of the 1964 Helsinki Declaration and its later amendments.

### 2.2. Participants

Thirty-five recreationally-active males between the ages of 18 and 35 years old were recruited for this study. Power analysis (G*Power V.3.1.9.4., Universität Düsseldorf, Düsseldorf, Germany) revealed that a minimum sample size for a repeated-measures within-factors design using one group, five positions, a power of 0.80, an α-value of 0.05, a nonsphericity correction of 1, a correlation among repeated measures of 0.5, and an effect size of 0.2 resulted in a sample size of 32. Participants were instructed to maintain normal dietary and exercise habits while enrolled in the study. Following an explanation of all procedures, risks, and benefits, each participant provided their written informed consent to participate on day 1 (T1). All participants were required to be healthy and free of any physical limitations (as determined by the MHAQ and PAR-Q+) and were deemed as resistance-trained, having participated in resistance training at least three times per week for at least the previous year. Participants were required to be non-smokers and be free from current and previous use of any performance-enhancing drugs. Participants were also excluded from the investigation if they were physically inactive, had a chronic illness causing the individual to seek medical care, had a pacemaker, were an amputee, or were unable to complete any of the exercise assessments on the familiarization day. All performance assessments and ultrasounds were performed on the dominant leg, which was designated during familiarization by each participant. 

One participant withdrew from the investigation after the familiarization day due to reasons unrelated to the study. Three participants were removed from the final data analysis due to issues related to ultrasound image analysis and performance measurements. Therefore, a total of 31 participants were included in the final analysis. Participant data is included in Table 1.

### 2.3. Familiarization Procedures

During T2, participants were familiarized with all performance assessments, which included unilateral vertical jump (UVJ), unilateral isometric and isokinetic knee extensions, and unilateral leg press. Prior to all physical performance assessments, participants were required to complete a standardized dynamic warm-up including: pedaling on a cycle ergometer for 5 min at a self-selected pace, 10 body-weight squats, 10 body-weight walking lunges, 10 dynamic walking hamstring stretches, 10 dynamic walking quadriceps stretches, 10 squat jumps, 10 arm circles, and 10 arm swings. 

For the UVJ familiarization, participants were instructed to perform a maximal unilateral countermovement jump with the dominant leg on a force plate (AccuPower, AMTI Watertown, MA, USA, 1000 Hz). Participants were instructed to stand on the dominant leg and flex the free leg at the knee, while keeping their hands placed on their hips throughout the duration of the UVJ. The joint angle during the UVJ was not standardized, however participants were instructed to squat down to a depth that allowed for maximum jump height on the concentric part of the jump. Participants were instructed on proper landing mechanics (e.g., no tucking) as not to affect flight time. To decrease the risk of injury, participants were instructed that they could land on two legs. A series of maximal UVJ were completed until the participant felt comfortable with the exercise. 

For the isokinetic and isometric knee extension assessments, participants were seated in an isokinetic dynamometer (S4, Biodex Medical System, Inc., New York, NY, USA), strapped into the chair at the waist, shoulders, and across the thigh to complete a series of isometric and isokinetic strength assessments. Chair and dynamometer settings were adjusted for each participant to properly align the axis of rotation with the lateral condyle of the femur. All participants were tested on their dominant leg, which was secured to the dynamometer arm just above the medial and lateral malleoli. The range of motion was assessed for each participant, and isokinetic dynamometer settings for each participant were consistent throughout testing. Participants first completed isometric and isokinetic warm-up sets at 50% of their perceived maximum. The isometric warm-up sets were performed while the knee remained positioned at an angle of 110°. The isometric warm-up sets consisted of three consecutive repetitions of leg extension, which incorporated 10 s of contraction, with a 10 s rest in between each repetition. Participants then completed an isokinetic warm-up, consisting of three sets of three isokinetic contractions of the knee extensor muscles at different angular velocities (60°·s^−1^, 180°·s^−1^, and 240°·s^−1^). Each isokinetic set consisted of concentric knee extension and passive knee flexion. A 60 s rest period was provided between each set, and 3 min of rest were provided after the last set. Participants were then instructed to perform two maximal voluntary isometric contractions (MVIC) at a knee angle of 110°, which was held for 6 s. Additionally, participants then performed three sets of three isokinetic contractions at different angular velocities (60°·s^−1^, 180°·s^−1^, and 240°·s^−1^). Participants were required to achieve an acceptable range of motion (~90°–170°) from to knee flexion to extension for each contraction. Between MVIC and isokinetic testing sets, 3 min of rest were provided to each participant.

For the unilateral leg press assessment, participants were seated in a unilateral leg press machine (Uni/Bi-Lateral Leg Press, PowerLift, Jefferson, IA, USA) and were familiarized with the unilateral leg press assessment. Participants were provided with proper instruction and technique for optimal exercise form and were instructed to complete unilateral leg presses with the dominant leg only. The seat position on the leg press apparatus was kept consistent for each participant for all testing days. Due to the unfamiliarity of a 1-RM unilateral leg press, participants underwent a 3-RM protocol on T2, which was then used to predict their 1-RM for T3. Each participant performed three warm-up sets before attempting a 3-RM lift. Following each warm-up set, additional weight was added to the leg press based upon the subject’s perceived difficulty. Repetition ranges for each of the three warm-up sets were 8–10 repetitions, 6–8 repetitions, and 4–6 repetitions, followed by 1, 2, and 3 min rest periods, respectively. Following the warm-up sets, additional weight was added to the leg press, and a 3-RM was attempted. Two to four subsequent trials were performed to determine each participant’s 3-RM. Trials not meeting the range of motion criteria (90° of knee flexion) for each exercise were discarded. Each participant’s 1-RM was then predicted using Equation (1) [38] for use during T3:

(1) $$1-RM=\frac{Weight}{1.0278-\left(0.0278\times \;Number\;of\;Repetitions\right)}$$

### 2.4. Testing Day Procedures

After at least 72 hours, participants returned to the laboratory for their testing visit (T3), which consisted of a hydration status assessment, anthropometric and body composition testing, ultrasound assessments, and physical performance testing. Participants were instructed to wear loose-fitting shorts during T3 to avoid compression of the upper leg musculature. All participants were required to have refrained from vigorous lower-body exercise for 72 h prior to the testing visit and from consuming alcohol and caffeine for at least 24 h prior to the testing visit. Participants were required to arrive for T3 in a hydrated state and having been fasted for a period of 4 h. A standardized snack (total energy: 260 kcal; carbohydrates: 48 g; protein: 3.1 ± 0.7 g; fat: 6 g) was provided to all participants after the ultrasound assessments and before physical performance assessments were completed.

#### 2.4.1. Hydration Status Assessment

Hydration status was determined upon arrival for testing at T3. Each participant was asked to provide a urine sample in a sterile container. Urine samples were analyzed for hydration status via refractometry by placing a drop of urine on a refractometer (Human Urine Refractometer, MISCO Refractometer, Cleveland, OH, USA) and digitally inspecting its osmolarity. Participants were considered euhydrated if the urine specific gravity was less than or equal to 1.020. If the participant was not adequately hydrated at the time of assessment, they were asked to drink water and were not permitted to continue until properly hydrated.

#### 2.4.2. Anthropometric and Body Composition Assessments

After the participant was confirmed to be in a state of euhydration, they were asked to remove their footwear, socks, and jewelry. Body mass (±0.1 kg) and height (±0.1 cm) were assessed using a Health-O-Meter Professional scale (Patient Weighing Scale, Model 500 KL, Pelstar, Alsip, IL, USA). Body composition (percent body fat, fat-free mass) was assessed via multi-frequency bioelectrical impedance analysis (BIA) machine (InBody770, InBody, Cerritos, CA), as previously described [39]. 

#### 2.4.3. Ultrasound Assessments

**Figure 2:**
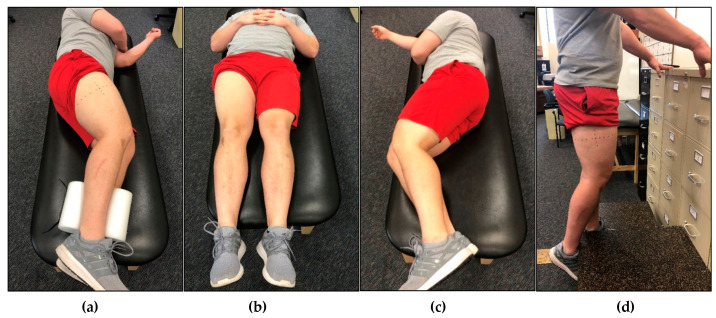
An example participant in different rest positions prior to ultrasound analysis of the vastus lateralis (VL) muscle. (**a**) Participant laying in the non-dominant lateral recumbent (NDLR) position with the dominant limb exposed. This was the position that all ultrasound images were captured in (except for standing), however, the rest position utilized beforehand differed. The participant utilized this rest position for immediately post (IP) analysis and rest for 15 min in the NDLR position. (**b**) Participant laying in the supine (SUP) position. After 15 min in this position, the participant was instructed to flip over to the NDLR position, and ultrasound images were captured immediately following. (**c**) Participant laying in the dominant lateral recumbent (DLR) position with the dominant leg compressed. After 15 min in this position, the participant was instructed to flip over to the NDLR position, and ultrasound images were captured immediately following. (**d**) Participant in the standing (ST) position. The ultrasound images were captured while the participant remained standing. Participants were instructed to bear weight on the non-dominant leg while resting the dominant leg against a platform to allow for a bend in the knee. Participants were instructed to stand for 15 min in between each position.

Each participant underwent five rounds of non-invasive ultrasound assessment of the VL in the dominant leg. Ultrasound rest positions are depicted in Figure 2a–d. For all lateral recumbent assessments, the participant’s legs were positioned to allow a 10° bend in the knees, as measured by a goniometer. Participant’s legs were stacked together, and a foam pad was placed between their ankles. For the first assessment, participants were positioned in the non-dominant lateral recumbent position (NDLR) (Figure 2a). Ultrasound images of the VL were captured immediately after the participant was positioned (IP). The assessments completed during IP took, on average, 113.9 ± 12.6 s to complete. Following IP, participants were instructed to remain in the NDLR position for 15 min, after which, additional ultrasound images were captured in the NDLR position. Following this, each participant was asked to stand for 15 min, and then asked to lay supine (SUP) on an examination table for 15 min (Figure 2b). Participants were then placed back in the NDLR position, and ultrasound assessments were taken immediately after the transition. Participants were again asked to stand for 15 min, and then asked to lay on an examination table in the dominant lateral recumbent (DLR) position for 15 min (Figure 2c). Participants were then placed back in the NDLR position, and ultrasound assessments were taken immediately after the transition. Participants were again asked to stand for 15 min, before being asked to stand on an elevated platform to obtain standing (ST) ultrasound images. While in the ST position, participants were instructed to bear weight only on their non-dominant limb, while the shin of the dominant limb rested against a higher platform to allow for a 10° bend in the knee (Figure 2d). Participants were instructed to completely relax the dominant leg against the higher limb to avoid muscle contraction of the VL. Ultrasound images were captured while participants remained in the standing position (ST), and were identical to those used during the recumbent positions. The order of all assessments except ST were randomized for each participant.

The ultrasound imaging techniques utilized in this investigation to assess the VL muscle have been previously described [26,27,28,29,30,31,32,33,34]. However, due to the primary research question of this investigation, the rest position that was utilized prior to ultrasound image capture was altered to examine the effects of the rest position on ultrasound characteristics. All anatomical locations of interest were identified using standardized landmarks for the VL muscle in the participants’ self-reported dominant limb. The landmarks for the VL were identified along the longitudinal distance over the femur at 50% of the distance from the greater trochanter to the lateral border of the patella [31,34]. To ensure proper probe placement and consistent image capture location, a semi-permanent marker was used to draw a dotted line transversely and longitudinally along the surface of the skin at the aforementioned location. The anatomical measurements for the VL were taken prior to anthropometric measurements. All measures of muscle morphology were obtained using a B-mode, 12-MHz linear probe (General Electric LOGIQ P5, Wauwatosa, WI, USA), coated with transmission gel (AquasonicVR 100, Parker Laboratories, Fairfield, NJ, USA) to provide acoustic contact without depressing the dermal layer of the skin [31,34]. Ultrasound settings remained fixed for examination of each participant to minimize instrumentation bias, to optimize spatial resolution, and to ensure consistency [31,34]. Image gain was set at 50 dB, dynamic range was set at 72, and image depth was set at 5 cm. Ultrasound images were captured in the transverse and sagittal planes, utilizing panoramic and still imaging. For each round of assessment, three panoramic images were captured in the transverse plane, perpendicular to the long axis of the muscle. Extended-field-of-view ultrasonography (LogiqView™) was used to capture panoramic images, which utilized a sweep of the probe along the VL from the medial portion of the muscle to the lateral portion of the muscle in order to capture the entire area of the muscle in a single image. Additionally, three still images were captured in the sagittal plane, parallel to the long axis of the muscle [33]. All ultrasound assessments were performed by the same examiner and were captured from the same anatomical locations. All ultrasound images were analyzed offline by an experienced researcher using image analysis software (ImageJ, National Institutes of Health, Bethesda, MD, USA, version 1.45s). A known distance shown in each ultrasound image was used to calibrate the image analysis software. Cross-sectional area (CSA), Subcutaneous fat thickness (SFT), uncorrected echo intensity (UnCorEI), corrected echo intensity (CorEI), muscle thickness (MT) and pennation angle (PA) of the VL were assessed for each rest position by the same technician that took the ultrasound images using the following procedures:

CSA of the VL was quantified using panoramic images captured in the transverse plane. The outline of the VL was located in each image and was traced using the polygon function tool in ImageJ, ensuring to include as much lean tissue as possible without including any surrounding bone or fascia [34]. The total area of each traced polygon was then calculated and reported in square centimeters. The average CSA of the three images taken in each rest position was then used for further analysis. 

UnCorEI was quantified within the region of interest previously demarcated for CSA determination. UnCorEI of the traced polygon was determined using the standard histogram function in ImageJ. Quantification of the grayscale of each individual pixel in the region of interest was expressed as a value between 0 and 255 arbitrary units (AU) (0: black; 255: white) [9,31,34]. The grayscale of each individual pixel was then projected on a histogram plot, and UnCorEI was quantified as the mean grayscale of the entire region of interest [9,31,34]. The average UnCorEI of the three images taken in each rest position was then used for further analysis.

In order to examine the potential influence of SFT on echo intensity (EI), SFT superficial to the VL was assessed in the images previously used for CSA and UnCorEI quantification. As previously described, SFT is defined as the perpendicular distance between the inferior border of the epithelium and the superior border of the superficial aponeurosis [40]. Quantification of SFT was determined as the average SFT adjacent to the lateral, mid-line, and medial portions of the VL using the line tool in ImageJ and is reported in centimeters [40,41]. The average SFT of the three images taken in each rest position was then used for further analysis. 

UnCorEI values for each panoramic image were then corrected for SFT (averaged from the SFT at the medial, mid-line, and lateral portions of the muscle) using Equation (2) previously established by Young et al. [40]:

(2) Corrected EI=Uncorrected EI+(SFT × 40.5278)

The average corrected EI (CorEI) values of the three images taken in each rest position was then used for further analysis.

MT was assessed using still images captured in the sagittal plane. MT was measured as the perpendicular distance from the superficial aponeurosis to the deep aponeurosis [29]. The MT was quantified using the straight-line tool in ImageJ at 50% of the horizontal distance of the image length and was reported in centimeters. The average MT of the three images taken in each rest position was then used for further analysis.

PA was assessed using the same images that were used for MT quantification. PA is defined as the angle of the intersection of the fascicles with the deep aponeurosis. PA was quantified using the angle tool in ImageJ and is reported in degrees (°). PA of three fascicles was measured in each image, and the average of the three were used for that image. The average PA of the three images taken in each rest position was then used for further analysis. 

Inter-day reliability for the quantification of CSA, UnCorEI, SFT, CorEI, MT, and PA of the VL using ultrasonography following rest in the SUP position were completed by the same ultrasound technician on a separate sample of participants, with at least 24 h between examinations. The intraclass correlation coefficient using model “3,1” (ICC_3,1_), SEM, minimal difference (MD), and coefficient of variation (CV) were calculated for each morphological variable: CSA: ICC_3,1_ = 0.997; SEM = 0.423 cm^2^; MD = 1.173 cm^2^; CV = 1.027%; UnCorEI: ICC_3,1_ = 0.935; SEM = 3.679 AU; MD = 10.199 AU; CV = 5.509%; SFT: ICC_3,1_ = 0.999; SEM = 0.022 cm; MD = 0.061 cm; CV = 3.044%; CorEI: ICC_3,1_ = 0.980; SEM = 4.308 AU; MD = 11.942 AU; CV = 4.747%; MT: ICC_3,1_ = 0.995; SEM = 0.029 cm; MD = 0.081 cm; CV = 1.071%; PA: ICC_3,1_ = 0.998; SEM = 0.272°; MD = 0.754°; CV = 2.103%.

#### 2.4.4. Physical Performance Assessments

Following ultrasonography, participants completed a standardized dynamic warm-up including: pedaling on a cycle ergometer for 5 min at a self-selected pace, 10 body-weight squats, 10 body-weight walking lunges, 10 dynamic walking hamstring stretches, 10 dynamic walking quadriceps stretches, 10 squat jumps, 10 arm circles, and 10 arm swings. Participants then completed the same performance assessments as during familiarization. All physical performance assessments were administered to each participant by the same researcher. Instructions for each assessment were provided to the participants through reciting a script. Verbal encouragement was given during each physical performance assessment. All assessments were supervised by a Certified Strength and Conditioning Specialist (CSCS) through the National Strength and Conditioning Association (NSCA).

For the UVJ assessment, participants were instructed to stand on the force plate on their dominant leg, with their hands placed on their hips. Participants performed a total of three maximal UVJ, with 3 min of rest between each jump. Participants were instructed that they could land on two feet if they preferred. Flight time was calculated as the time interval from toe-off to landing, and UVJ height was calculated using flight time. Furthermore, PF was measured, and peak power, the rate of power development (RPD), total work, and peak velocity were calculated for each UVJ. The greatest values for each variable from the three UVJ were then used for further analysis. 

Following the UVJ assessment, participants underwent the same unilateral isometric and isokinetic testing protocol that was completed during the familiarization day. For each test, torque signals were sampled at 1 kHz with a data acquisition system (MP150 BIOPAC Systems, Inc., Santa Barbara, CA, USA), recorded on a personal computer, and processed offline. For each MVIC, a torque-time curve was created. Due to the influence of dynamometer arm length on torque, a correction was applied to the torque values to independently examine the effects of muscle morphology in different rest positions on isometric and isokinetic force. The torque values obtained from the isokinetic dynamometer were divided by the dynamometer arm length setting of the Biodex for each participant to account for the influence of moment arm on torque (Equation (3)):

(3) $$Force=\frac{Torque}{Moment\;Arm}$$

Therefore, muscle force production was examined after accounting for dynamometer arm setting length. For each MVIC, the onset of torque was determined when the torque signal crossed the value equal to 10% above the baseline. PF, RFD over 50 ms (RFD50), 100 ms (RFD100), 200 ms (RFD200), and impulse over 50 ms (IMP50), 100 ms (IMP100), and 200 ms (IMP200) were recorded for each MVIC. PF was identified as the greatest force achieved on the force-time curve for each repetition. RFD was defined as the greatest rate of change of force development over time between sampled data points. IMP was defined as the average force generated over time. For each set of isokinetic knee extension, PF was recorded: isokinetic PF at 60°·s^−1^ (IsokPF (60°·s^−1^)), isokinetic PF at 180°·s^−1^ (IsokPF (180°·s^−1^)), and isokinetic PF at 240°·s^−1^ (IsokPF (240°·s^−1^)). The greatest values for each variable was used for further analysis. 

In support of the correction for moment arm in the examination of muscle morphology and its relationship with force production, Biodex dynamometer arm length was found to be a significant predictor of performance on all isometric and isokinetic torque variables (peak torque (PT), rate of torque development (RTD) over 50 ms (RTD50), 100 ms (RTD100), 200 ms (RTD200), and impulse over 50 ms (IMP50), 100 ms (IMP100), and 200 ms (IMP200)), except RTD100 (Table 2). Unless the dynamometer length is accounted for, torque values obtained from isokinetic dynamometry testing may not reflect true muscle force-producing capabilities.

To determine the maximal strength of each individual, participants then completed a unilateral leg press assessment according to guidelines published by the NSCA. Each participant performed three warm-up sets before attempting a 1-RM lift. The external loads used during the warm-up sets and the 1-RM attempts were based off of the estimated 1-RM from the 3-RM determined during familiarization. Following each warm-up set, additional weight was added to the leg press based upon the subject’s perceived difficulty. Repetition ranges for each of the three warm-up sets were 8–10 repetitions, 4–6 repetitions, and 2–3 repetitions, followed by one-, two-, and three-minute rest periods, respectively. Following the warm-up sets, additional weight was added to the leg press, and a 1-RM was attempted. Two to four subsequent trials were performed to determine a 1-RM. Trials not meeting the range of motion criteria (90° of knee flexion) for each exercise were discarded. 

### 2.5. Statistical Analyses

Intra-examiner precision between three consecutive panoramic and still images captured from each subject was analyzed using the SEM for CSA, UnCorEI, CorEI, MT, PA, and SFT [33]. The CV and ICC_3,1_ for each muscle morphological characteristic were also calculated to establish reliability [42]. Additionally, the minimal difference (MD) was calculated for each muscle morphological characteristic, which refers to the minimum difference between values that reflect a true change. Prior to statistical procedures, all data was assessed for normality and sphericity. If the assumption of sphericity was violated, a Greenhouse–Geisser correction was applied. To analyze within-subject differences in ultrasound-derived morphological characteristics of the VL (CSA, UnCorEI, CorEI, MT, PA, SFT), a repeated-measures analysis of variance (ANOVA) was used. In the event of a significant interaction, least significant differences (LSD) post-hoc tests were used for pairwise comparisons. Rest position effects were further analyzed using partial eta squared (η_p_^2^). Interpretations of η_p_^2^ were evaluated in accordance with Cohen [43] at the following levels: small effect (0.01–0.058), medium effect (0.059–0.137), and large effect (>0.138). Comparisons between rest positions were further analyzed using 95% confidence intervals (CI) and Cohen’s *d*. Magnitudes of the standardized effects were interpreted using thresholds of <0.2, 0.2–0.6, 0.6–1.2, 1.2–2.0, 2.0–4.0. These values corresponded to trivial, small, moderate, large, and very large effect sizes (ES), respectively.

Associations between muscle morphological characteristics (CSA, UnCorEI, CorEI, MT, PA, and SFT) and physical performance variables were examined using Pearson’s *r*. Additionally, stepwise linear regression was used to assess the shared variance (*R*^2^) between muscle morphological characteristics and physical performance variables. Entry into the model occurred if the significance of the F value for a specific position was *p* < 0.05, and the independent variable with the highest correlation to the dependent variable was included into the regression equation. Correlation magnitudes were quantified using the following descriptors: 0.00–0.10: *trivial*; 0.11–0.30: *small*; 0.31–0.50: *moderate*; 0.51–0.70: *large*; 0.71–0.90: *very large*; 0.91–1.00: *almost perfect* [44]. For all analyses, a criterion alpha level of α ≤ 0.05 was used to determine statistical significance, and statistical software (Statistical Package for the Social Sciences [SPSS] V.23.0, Chicago, IL, USA) was used. All data are reported as mean ± standard deviation.

Differences between two dependent correlation coefficients with one variable in common were tested using the Williams modification of the Hotelling test [45]. This method was used to determine if one correlation was significantly different from another correlation with one common variable, using the following equation (Equation (4)):

(4) $$t\;\left(n-3\right)=\frac{\left(r_{12}-r_{23}\right)\sqrt{\left(n-1\right)\left(1+r_{12}\right)}}{\sqrt{2K\;\frac{\left(n-1\right)}{\left(n-3\right)}\;+\;\frac{{\left(r_{23}+r_{13}\right)}^{2}}{4}\;{\left(1-r_{12}\right)}^{3}}}$$

where:

$$K=1-r_{12}^{2}-r_{13}^{2}-r_{23\;}^{2}+2r_{12}r_{13}r_{23}$$

The two correlation coefficients to be compared (i.e., *r*_12_ and *r*_13_), along with the correlation coefficient between the two unshared variables (i.e., *r*_23_), and the sample size were inputted into a published spreadsheet (“Comparing Pairs of Correlations,” University of Cambridge, accessible at http://imaging.mrc-cbu.cam.ac.uk/statswiki/FAQ/WilliamsSPSS?action=AttachFile&do=view&target=Williams-test.xlsx). The *p*-value associated with a two-tailed test of significance was then computed. Results were considered significant at α ≤ 0.05.

## 3. Results

### 3.1. Ultrasound Assessments

Most of the ultrasound morphological variables exhibited normality, therefore, comparisons of mean differences in muscle morphological characteristics after rest in different positions were assessed using parametric analysis.

Reliability and precision values for all muscle morphological characteristics after rest in different positions are presented in Table 3. These results indicate high reliability and precision between images for each variable after rest in all positions; however, PA consistently provided the lowest reliability and precision values, regardless of rest position.

Values for ultrasound-derived muscle morphological characteristics between different rest positions are presented in Table 4. 

A significant main effect for rest position was observed for all muscle morphological variables (CSA: F_2.941, 88.238_ = 7.206, η_p_^2^ = 0.194, *p* < 0.001; UnCorEI: F_2.311, 69.345_ = 18.196, η_p_^2^ = 0.378, *p* < 0.001; CorEI: F_2.522, 69.345_ = 5.046, η_p_^2^ = 0.144, *p* = 0.005; MT: F_1.891, 56.723_ = 85.671, η_p_^2^ = 0.741, *p* < 0.001; PA: F_2.577, 77.322_ = 35.621, η_p_^2^ = 0.543, *p* < 0.001; SFT: F_1.978, 59.335_ = 12.660, η_p_^2^ = 0.297, *p* < 0.001). 

CSA was significantly greater in ST compared to NDLR (*p* < 0.001; *d* = 0.12; 95% CI = 0.442–1.147), SUP (*p* < 0.001; *d* = 0.12; 95% CI = 0.392–1.156), and DLR (*p* = 0.009; *d* = 0.10; 95% CI = 0.171–1.107), but was not different from IP (*p* = 0.070; *d* = 0.06; 95% CI = −0.036–0.861). Additionally, CSA was significantly greater in IP compared to NDLR (*p* = 0.010; *d* = 0.06; 95% CI = 0.099–0.665) and SUP (*p* = 0.007; *d* = 0.06; 95% CI = 0.106–0.617), but was not significantly different from DLR (*p* = 0.167; *d* = 0.04; 95% CI = −0.100–0.554).

UnCorEI was significantly lower in ST compared to all other positions: IP (*p* < 0.001; *d* = 0.50; 95% CI = −4.455–−2.057), NDLR (*p* < 0.001; *d* = 0.47; 95% CI = −4.176–−1.814), SUP (*p* < 0.001; *d* = 0.37; 95% CI = −3.577–−1.263), and DLR (*p* = 0.001; *d* = 0.30; 95% CI = −2.896–−0.805). Additionally, UnCorEI was significantly greater in IP compared to SUP (*p* = 0.017; *d* = 0.12; 95% CI = 0.163–1.509) and DLR (*p* < 0.001; *d* = 0.22; 95% CI = 0.789–2.021), but was not significantly different from NDLR (*p* = 0.359; *d* = 0.04; 95% CI = −0.310–0.831). UnCorEI was significantly greater in NDLR compared to DLR (*p* = 0.001; *d* = 0.18; 95% CI = 0.517–1.772). No differences were observed between NDLR and SUP (*p* = 0.092; *d* = 0.09; 95% CI = −0.100–1.250) or DLR and SUP (*p* = 0.083; *d* = 0.09; 95% CI = −1.218–0.079). 

CorEI was significantly lower in ST compared to IP (*p* = 0.019; *d* = 0.11; 95% CI = −3.141–−0.306), and NDLR (*p* = 0.037; *d* = 0.09; 95% CI = −2.590–−0.085), but was not significantly different from SUP (*p* = 0.983; *d* = 0.00; 95% CI = −1.258–1.231) or DLR (*p* = 0.649; *d* = 0.02; 95% CI = −1.544–0.976). Additionally, CorEI was significantly greater in IP compared to SUP (*p* = 0.001; *d* = 0.12; 95% CI = 0.721–2.700) and DLR (*p* = 0.001; *d* = 0.10; 95% CI = 0.670–2.209), but was not significantly different from NDLR (*p* = 0.182; *d* = 0.03; 95% CI = −0.190–0.963). CorEI was significantly greater in NDLR compared to SUP (*p* = 0.008; *d* = 0.09; 95% CI = 0.377–2.271) and DLR (*p* = 0.004; *d* = 0.07; 95% CI = 0.357–1.750). No significant differences in CorEI were observed rest in DLR compared to SUP (*p* = 0.510; *d* = 0.02; 95% CI = −0.557–1.099). 

MT was significantly greater in ST compared to all other positions: IP (*p* < 0.001; *d* = 0.99; 95% CI = 0.321–0.465), NDLR (*p* < 0.001; *d* = 0.97; 95% CI = 0.311–0.461), SUP (*p* < 0.001; *d* = 1.02; 95% CI = 0.322–0.475), and DLR (*p* < 0.001; *d* = 0.98; 95% CI = 0.322–0.481), but was not different between recumbent positions. 

PA was significantly greater in ST compared to all other positions: IP (*p* < 0.001; *d* = 1.17; 95% CI = 3.953–6.974), NDLR (*p* < 0.001; *d* = 1.22; 95% CI = 3.862–6.991), SUP (*p* < 0.001; *d* = 1.32; 95% CI = 4.357–7.300), and DLR (*p* < 0.001; *d* = 1.33; 95% CI = 4.491–7.917), but was not different between recumbent positions. 

SFT was significantly greater in ST than in all other positions: IP (*p* < 0.001; *d* = 0.13; 95% CI = 0.018–0.057), NDLR (*p* < 0.001; *d* = 0.14; 95% CI = 0.021–0.061), SUP (*p* < 0.001; *d* = 0.12; 95% CI = 0.019–0.053), and DLR (*p* < 0.001; *d* = 0.13; 95% CI = 0.020–0.057), but was not different between recumbent positions. 

### 3.2. Associations between Muscle Morphology and Physical Performance

All physical performance data exhibited normality except for UVJ RPD and leg press 1-RM. Associations between muscle morphological characteristics after rest in different positions and physical performance was assessed using parametric analysis due to a majority of the variables exhibiting normality.

#### 3.2.1. Unilateral Vertical Jump (UVJ) Performance

Associations between UVJ performance and ultrasound morphological characteristics are presented in Table 5. 

CSA was the best predictor of both total work and peak power in IP, and the best predictor of PF in DLR. The magnitude of relationship between CSA and total work and peak power in DLR was not different compared to other positions; however, the magnitude of relationship between CSA and PF was significantly greater in DLR compared to SUP.

CorEI was the best predictor of peak velocity in IP, and the best predictor of jump height in NDLR. The magnitude of relationship between CorEI and peak velocity in IP was not different compared to other positions, while the relationship between CorEI and jump height was *large* in all positions except ST. UnCorEI was not a significant predictor of any UVJ performance variable.

MT was the best predictor of PF in IP and peak power in ST. The magnitude of relationship between MT and PF in IP was not different compared to other positions, and the magnitude of relationship between MT and peak power in ST was not different compared to IP, NDLR, or DLR. No significant correlation between MT and peak power was observed for SUP. 

PA was the best predictor of jump height, peak power, peak velocity, and RPD in IP. The relationship between PA and jump height and peak velocity in IP were the only statistically significant relationship between these variables in any position. The relationship between PA in IP and jump height was significantly stronger than that in DLR and SUP, and the relationship between PA in IP and peak velocity was significantly stronger than that in SUP. The magnitude of relationship between PA and peak power in IP was not different compared to NDLR and DLR, while no significant correlations existed between PA and peak power in SUP or ST. The magnitude of relationship between PA and RPD in IP was not different compared to NDLR, DLR, and SUP however, this relationship was significantly stronger than that between PA and peak power in ST. Additionally, PA was the best predictor of PF in DLR, and the magnitude of this relationship was not different compared to IP, NDLR, or SUP; however, each of these positions provided significantly stronger relationships than that of PA in ST and PF. 

SFT was the best predictor of both jump height and peak velocity in IP. The magnitude of relationship between SFT in IP and jump height and peak velocity was not different compared to other positions.

#### 3.2.2. Unilateral Isometric and Isokinetic Performance

No significant associations existed between uncorrected isometric and isokinetic performance values and muscle morphological characteristics after rest in any position. 

UnCorEI was the only muscle morphological characteristic that provided a statistically significant relationship with any isometric variable after correcting for dynamometer arm length (table not shown). UnCorEI was the best predictor of IMP50 in IP, however the magnitude of this relationship was not different compared to SUP or ST, but was significantly stronger than the relationship between UnCorEI and IMP50 in NDLR. UnCorEI in NDLR and DLR were not significantly correlated with IMP50.

Associations between muscle morphological characteristics and isokinetic performance values after adjusting for dynamometer arm length with their best rest position predictor, along with their practical interpretation, are presented in Table 6. 

MT was the best predictor of both IsokPF (180°·s^−1^) and IsokPF (240°·s^−1^) in DLR. The magnitude of relationship between MT and IsokPF (180°·s^−1^) in DLR was not different compared to other positions, while the magnitude of relationship between MT and IsokPF (240°·s^−1^) was not statistically significant in SUP or ST. 

PA was the best predictor of both IsokPF (60°·s^−1^) and IsokPF (240°·s^−1^) in DLR, and no other rest positions provided statistically significant relationships between PA and either variable. PA was the best predictor of IsokPF (180°·s^−1^) in ST; however the magnitude of this relationships was not different compared to other positions. 

CSA, CorEI, and SFT were not significant predictors of any isometric or isokinetic variables after rest in any position.

#### 3.2.3. Unilateral Maximal Strength

Associations between maximal strength values and ultrasound-derived morphological characteristics are presented in Table 7. 

CSA, MT, and PA were significant predictors of maximal strength, but UnCorEI, CorEI, and SFT were not. CSA and PA were the best predictors of 1-RM unilateral leg press after rest in NDLR. CSA was significantly correlated with 1-RM after rest in all other positions, and the magnitude of these relationships were not significantly different from each other; however CSA evaluated in NDLR and IP provided very large relationships with maximal strength, whereas CSA in SUP, DLR, and ST exhibited only large relationships. PA exhibited moderate relationships with 1-RM after rest in all positions, but PA in ST was not significantly related to 1-RM. Additionally, MT in IP exhibited very large relationship with 1-RM in IP, whereas MT in all other positions provided large relationships. MT in IP explained more of the variance (52.2%) in unilateral maximal strength compared to either CSA (50.8%) or PA (18.2%) in NDLR.

## 4. Discussion

Measurements of muscle morphology in the present study demonstrated high precision in all positions measured, although PA consistently provided the lowest values, regardless of position. The main findings of this study suggest that CSA, UnCorEI, and CorEI of the VL differ significantly between NDLR, DLR, and SUP; however, MT, PA, and SFT appear to remain consistent. Additionally, CSA, MT, PA, and SFT were significantly greater in ST compared to NDLR, DLR, and SUP. Muscle morphology measured in the IP position appears to be best related to physical performance, although utilizing the NDLR and DLR positions may provide comparable results. Although standing measures of VL morphology have previously been reported to exhibit stronger relationships with performance than recumbent measures [15], our results suggest that the relationship between muscle morphology and performance in ST may be weaker compared to the recumbent positions.

**Figure 3:**
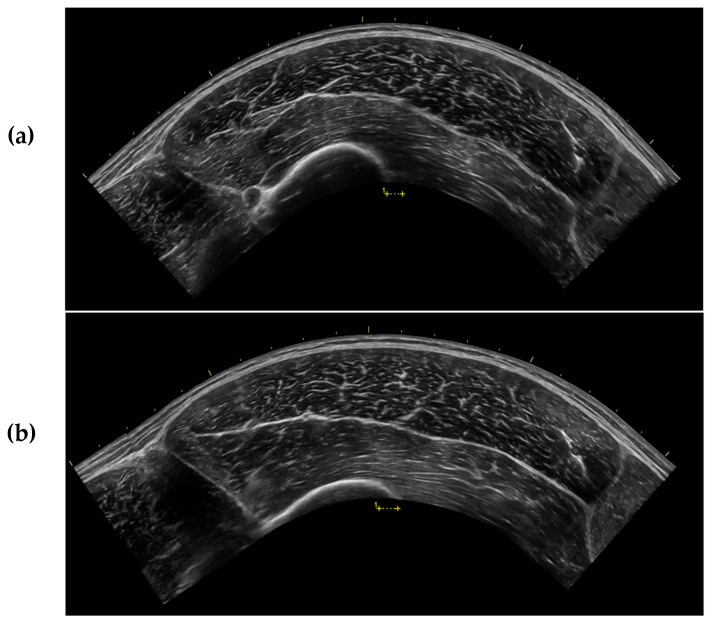
Sample ultrasound images captured from the same participant after 15 min of rest in (**a**) the supine position (SUP) and (**b**) during standing (ST). All ultrasound settings were kept consistent throughout testing for each participant.

The current investigation demonstrated that MT of the VL was significantly greater in ST compared to all recumbent positions, and CSA was significantly greater in ST compared to all recumbent positions except IP, although a trend was observed between IP and ST. These findings align with those of Wagle et al. [15], indicating that measurements of muscle size taken at the muscle belly may be influenced by changes in position in the absence of changes in CSA. This may be a result of muscle “gearing” where muscle fibers shorten in the longitudinal direction and expand in the transverse direction. This causes the muscle fibers to rotate to a greater PA, which creates a bulging effect in the center of the muscle [46,47]. Consistent with this, we observed a significantly greater PA in ST compared to all other positions. Nevertheless, muscle gearing is typically reported during muscle contraction, when a change in the length of the muscle is induced [46]. In the present study, careful consideration was taken to ensure that joint angle remained constant and the muscle was relaxed in all positions examined. It is apparent that changes in position can create a similar muscle-bulging effect due to the influence of gravity on muscle shape and size [14,25] that may not be due to true muscle gearing. Anecdotally, we noted that the VL appeared flatter and longer in the recumbent positions compared to ST, which may have allowed for only modest changes in CSA as compared to the larger changes in MT [25] (Figure 3).

In the present study, we observed a significant decrease in VL CSA after 15 min of rest in the recumbent position (from IP to NDLR), with no significant change in MT, which is consistent with the findings of others [22,24]. Arroyo and colleagues [22] observed a significant decrease in VL CSA between 0 and 10 min after transitioning from standing to recumbent positions in the absence of changes in MT, while Shea [24] observed a similar decrease in VL CSA following 20 and 30 min of supine rest. These findings suggest that fluid shifts may impact VL CSA to a greater degree than MT, whereas MT appears to be influenced more by changes in posture (from ST to IP) and muscle bulging. These findings are in contrast to those of Lopez et al. [23] and Tomko et al. [25] who observed no change in rectus femoris (RF) CSA after transitioning from a standing to a supine or seated position, respectively. Speculatively, the larger size and different structure of the VL compared to the RF may allow for a larger quantity of fluid shifting into and out of the muscle, or perhaps within the muscle. Additionally, the present study and the work of Arroyo et al. [22] utilized young, healthy adults, whereas Lopez et al. [23] utilized older adults. With aging, a decrease in contractile tissue along with an increase in the relative proportion of intramuscular fat and fibrous tissue is often observed. This may lead to a decrease in fluid storage within the muscle [48], which may lessen the likelihood of fluid shifts in response to changes in posture. Consistent with this, Cerniglia and colleagues [11] observed no change in the CSA of mid-thigh low-density muscle after 15 min of supine rest, while Shea [24] reported no changes in CSA of the VL in individuals who engaged in low amounts of physical activity until between 20 and 30 min following recumbency. Nevertheless, Tomko et al. [25] observed no change in RF CSA in physically-active, young males and females. However, CSA was only measured only five minutes after changing position. Although fluid shifts have been shown to occur rapidly upon changes in posture [13,17,49,50], these changes may not become evident within five minutes of position change [22]. Additionally, participants in the Tomko et al. [25] investigation transitioned from a seated to supine position, which results in smaller fluid shifts than transitioning from the supine to standing position [13,51].

CSA was significantly lower in NDLR compared to IP in the present study, and in SUP compared to IP; however, no differences were observed between IP and DLR. These findings may indicate that rest in the DLR position may minimize fluid shifts in the dominant limb. Since hydrostatic pressure within a body part is based on its vertical height from the heart [52], transitioning from a supine to lateral recumbent position alters the positioning of the dominant leg relative to the heart, which may alter blood flow. In the DLR position, an increased hydrostatic pressure and accumulation of blood will occur in the vessels on the dominant side of the body [35,36], which may allow for a greater accumulation of fluid in the tissues, resulting in a slightly larger CSA. 

In the present study, we observed a significantly lower UnCorEI in ST compared to all recumbent positions. Previous research suggests that an attenuation of ultrasound waves may occur in tissues that are examined at a greater depth, which may artificially decrease EI values in deeper tissues [9,40]. With a postural change from standing to recumbency, it appears that SFT may decrease due to fluid shifts out of the subcutaneous layer [10,53]. This aligns with our findings where SFT values were greater in ST relative to all recumbent positions. The greater SFT may have contributed to a greater overall depth of the muscle, which may account for the decreased UnCorEI. However, a postural change from the ST to SUP position also results in fluid shifts from the muscle, which would likely result in a lower muscle water content, and a pursuant increase in UnCorEI [9]. In the present study, when correcting for SFT, CorEI values in ST remained significantly lower than those during IP and NDLR but were not different from SUP and DLR. These findings indicate that small, nonsignificant changes in SFT may have large implications for CorEI. 

The present study demonstrated that UnCorEI did not change after 15 min of rest in the recumbent position (from IP to NDLR), which aligns with the findings of others [22,23] in the VL. In contrast, Shea [24] observed significant increases in UnCorEI of the VL in a sample of younger and older individuals after transitioning from a standing to recumbent position, which was followed by a subsequent decline back to original values. However, when correcting for SFT, CorEI and SFT values in younger individuals did not change over time [24]. In alignment, we observed no change in SFT after 15 min of rest in any recumbent position, which may explain the lack of change in both UnCorEI and CorEI over time (from IP to NDLR). 

Both CorEI and UnCorEI differed between recumbent positions. The UnCorEI values were significantly lower in DLR and SUP as compared to IP, and in DLR as compared to NDLR, while CorEI values were significantly lower in DLR and SUP as compared to IP and NDLR. Previous research has suggested that fluid shifts and water content of the muscle affect UnCorEI [9]. A decrease in CSA of the muscle as a result of gravitational fluid shifts may hypothetically also accompany an increase in UnCorEI [9]. Of the three measurements taken in the recumbent positions after 15 min of rest (NDLR, SUP, and DLR), we reported that DLR exhibited the lowest UnCorEI and the greatest CSA. However, the greatest EI (both UnCorEI and CorEI) was observed in IP, which had the greatest CSA of all of the recumbent positions. These findings were unexpected, especially considering that the IP measurements were taken immediately after transitioning from a standing to recumbent position, and ST had the lowest UnCorEI and CorEI values. Previous research has reported that UnCorEI may increase within the first five minutes after changing from a standing to recumbent [24] or seated [25] position, followed by a decline over time [25]. Further research is necessary to elucidate these findings, but based on the current study and others [22,23,24,25], the association between changes in muscle EI and muscle size with postural shifts may not be linear. Additionally, changes in muscle EI may not directly reflect absolute changes in muscle fluid shifts, but rather may be more sensitive to the rate of change in fluid within the muscle [23], SFT [24], or probe handling [54]. 

In the present investigation, both CSA and MT were significantly correlated with UVJ PF and peak power, as well as 1-RM leg press. However, only MT was significantly correlated with IsokPF, and only CSA was significantly correlated with UVJ total work. Although measures of MT and CSA appear to be highly correlated [33], researchers have suggested that increases in lower-body strength may be better elucidated by MT rather than changes in CSA and, therefore, quantification of both may be necessary for a comprehensive understanding of muscle function [34]. However, neither CSA nor MT was a significant predictor of UVJ height, peak velocity, or RPD in the present study, which is consistent with research indicating that peak velocity and RPD are related primarily to fiber orientation, fiber-type distribution, and efferent neural drive rather than muscle size [55,56,57,58,59]. In the present study, PA was significantly associated with all jump variables except for total work, as well as all isokinetic variables, and 1-RM leg press, which is consistent with the notion that fiber arrangement affects both maximal strength as well as RFD. 

In addition to muscle size and fiber orientation, EI has been shown to be related to force production [28,29,31,60,61]. In the present investigation, CorEI was significantly correlated with UVJ height and peak velocity, whereas UnCorEI was not a significant predictor of any UVJ variable, which may indicate that CorEI may be preferred over UnCorEI when examining jump performance. Notably, SFT had comparable shared variance to CorEI for predicting jump variables. However, neither CorEI nor UnCorEI was associated with isometric performance or 1-RM leg press.

In contrast to previous research stating that stronger relationships may be observed between standing measures of muscle morphology and performance [15], the majority of performance variables measured in the present investigation demonstrated greater relationships with recumbent measures. A potential explanation for the discrepancy in these findings is that the types of performance tests administered in each study differed: the tests in the study of Wagle et al. [15] were all conducted in the upright position, whereas in the present investigation, the UVJ was conducted in the upright position, the isometric/isokinetic measures were completed while the participant was seated, and the 1-RM leg press was completed while the participant was in a reclined seated position. Wagle et al. [15] suggest that the ability of muscle morphology to predict performance may be a factor of how the muscle is analyzed relative to the position in which the muscle is utilized. Therefore, measurements taken during ST may reflect muscle function only during upright activities. However, in the current study, ST was the greatest predictor of only UVJ peak power through MT. Further, ST measurements of CorEI provided only moderate relationships with UVJ height, whereas CorEI in all other positions provided large relationships with UVJ height. The ST position did not provide significant correlations with UVJ height, PF, or peak velocity; however, PA in select recumbent positions did. In the seated position, no measure of morphology in ST was a significant predictor of IsokPF (240°·s^−1^), although other measures in recumbent positions were. 

Wagle et al. [15] reported stronger relationships between standing measures of muscle size and isometric variables compared to supine measures, however, in the current study, no measures of muscle size were significantly related to any of the isometric variables. This may be a result of a discrepancy between positions, as the ultrasound assessments were completed while in the recumbent position or while standing and the isometric tests were completed while seated. Additionally, previous research suggests that changes in muscle length (as induced by a seated position in comparison to a supine position) has a considerable effect on increasing knee extension torque because of the additional involvement of the RF muscle [62]. Therefore, the isometric and isokinetic variables examined in this investigation may have been better elucidated by examining both RF and VL morphology. Further, all strength assessments in the investigation of Wagle et al. [15] involved the use of both limbs, but only right VL morphology was assessed, whereas the present investigation examined both muscle morphology and performance in the dominant limb. 

In general, morphology of the VL assessed after IP appears to be the best predictor of physical performance. All muscle morphological characteristics that were significant predictors of UVJ performance and 1-RM leg press included IP as a rest position. Although IP provided comparable relationships with UVJ performance variables to DLR and NDLR, PA after rest in IP was the only significant predictor of UVJ height and peak velocity, and MT in IP provided very large relationships with 1-RM leg press, whereas the other positions provided large relationships. Additionally, although PA after rest in DLR was the only significant predictor of IsokPF (60°·s^−1^) and IsokPF (240°·s^−1^), the strength of these relationships were not different from those provided by IP. Therefore, it is evident that waiting for fluid shifts to occur prior to ultrasound assessment of the VL may not be necessary when predicting performance and, instead, may rather diminish the ability of muscle morphological characteristics to predict function [11,22]. 

Notably, VL morphology taken after rest in SUP was not the best predictor of any of the performance variables. As this is typically the rest position utilized in most previous reports of ultrasonography, [27,28,30,31,32,33,34], future investigations may want to avoid using this position prior to ultrasound assessment in order to obtain the best prediction of VL muscle function. 

Although we attempted to recruit a relatively homogenous sample of participants for this investigation, our results may be limited to physically-active young males, and future research is necessary to determine if similar changes in muscle morphology after rest in different positions are observed in other populations. Additionally, many of our performance variables were not significantly correlated with muscle morphological characteristics in any position, which may be viewed as a limitation in this study. As muscle strength is dependent on the combination of various internal and external factors (i.e., neural control, motivation, etc.), the evaluation of muscle morphology does not fully explain a muscle’s force-producing capabilities. Furthermore, all physical performance assessments were performed on only one leg, which may have been a novel stimulus for the participants. However, we attempted to diminish any potential learning effect of these new techniques through the inclusion of a familiarization session with each performance assessment.

## 5. Conclusions

In conclusion, VL CSA, UnCorEI, and CorEI differ after rest in different recumbent positions; however, MT, PA, and SFT appear to remain consistent. All measures of muscle morphology in ST are different from those obtained after 15 min of rest in the recumbent positions, except for CorEI. Muscle morphology in IP most consistently provides the greatest relationship with unilateral lower-body performance but may be comparable to NDLR and DLR. Additionally, some measures of muscle morphology in ST and SUP provided significantly weaker relationships with performance variables compared to other recumbent positions. Thus, researchers and practitioners that aim to use ultrasonography to assess muscular force and power of the lower-body should consider evaluation of muscle morphology of the vastus lateralis immediately after laying down to best predict performance instead of using 15 min of rest in SUP, as typically prescribed. The positioning of the body on an examination table also necessitates fewer requirements on the technician and the subject and, therefore, may be preferred over standing ultrasounds in many settings.

The findings of this investigation may also have important implications for clinicians and practitioners using ultrasonography to evaluate muscle structure and function. The results of this study highlight the importance of standardization of a rest position prior to ultrasound examination and demonstrate that rest in an unspecified recumbent position will result in discrepancies in muscle measurements. Additionally, these findings underline the possibility that similar changes in size and structure of other bodily tissues may occur when resting in different positions, which may have crucial implications in detection and diagnosis of clinical conditions and pathologies. Further research is necessary to determine whether changes in rest position affect other bodily tissues to the same extent as muscle.

## Figures and Tables

**Table 1:** Demographic and anthropometric measurements of participants included in the final data analysis.

*N*	Age (yrs)	Height (m)	Body Mass (kg)	Body Fat (%)
31	23.0 ± 2.1	1.79 ± 0.08	87.4 ± 11.7	18.0 ± 5.2

Values are presented as mean ± standard deviation.

**Table 2:** Associations between Biodex dynamometer arm length setting and unilateral isometric and isokinetic knee extension performance variables.

Biodex Variable	*r*	*p*-Value
MVIC PT	0.400	0.026 *
MVIC RTD50	0.471	0.008 *
MVIC RTD100	0.344	0.058
MVIC RTD200	0.364	0.044 *
MVIC IMP50	0.588	0.001 *
MVIC IMP100	0.492	0.005 *
MVIC IMP200	0.472	0.007 *
IsokPT (60°·s^−1^)	0.496	0.005 *
IsokPT (180°·s^−1^)	0.429	0.016 *
IsokPT (240°·s^−1^)	0.504	0.004 *

*r*: Pearson’s correlation coefficient; MVIC: Maximal voluntary isometric contraction; PT: Peak torque; RTD50: Rate of torque development over 50 ms; RTD100: Rate of torque development over 100 ms; RTD200: Rate of torque development over 200 ms; IMP50: Impulse over 50 ms; IMP100: Impulse over 100 ms; IMP200: Impulse over 200 ms; IsokPT (60°·s^−1^): Isokinetic peak torque at 60° per second; IsokPT (180°·s^−1^): Isokinetic peak torque at 180° per second; IsokPT (240°·s^−1^): Isokinetic peak torque at 240° per second. *Statistically significant correlation (*p* ≤ 0.05).

**Table 3:** Reliability and precision values for ultrasound-derived morphological characteristics of the vastus lateralis after rest in different positions.

Rest Position	Variable	ICC_3,1_	CV (%)	SEM	MD
IP	CSA	0.996	1.054	0.404	1.120
	UnCorEI	0.976	2.316	1.059	2.934
	CorEI	0.994	1.162	1.197	3.317
	MT	0.990	1.643	0.045	0.123
	PA	0.811	11.216	2.270	6.293
	SFT	0.996	2.826	0.018	0.049
NDLR	CSA	0.995	1.186	0.486	1.346
	UnCorEI	0.967	2.186	1.206	3.342
	CorEI	0.992	1.594	1.376	3.815
	MT	0.988	1.735	0.049	0.135
	PA	0.861	8.838	1.752	4.859
	SFT	0.997	2.235	0.015	0.043
SUP	CSA	0.995	1.300	0.473	1.310
	UnCorEI	0.957	2.947	1.444	4.004
	CorEI	0.988	2.072	4.512	1.628
	MT	0.989	1.787	0.044	0.123
	PA	0.857	9.358	1.755	4.865
	SFT	0.998	2.111	0.014	0.038
DLR	CSA	0.972	1.515	1.081	2.996
	UnCorEI	0.940	3.216	1.552	4.301
	CorEI	0.989	2.157	1.525	4.227
	MT	0.991	1.739	0.044	0.123
	PA	0.911	8.570	1.511	4.187
	SFT	0.998	2.303	0.015	0.041
ST	CSA	0.995	1.205	0.494	1.369
	UnCorEI	0.959	2.784	1.309	3.629
	CorEI	0.994	1.666	1.210	3.354
	MT	0.970	2.302	0.063	0.174
	PA	0.801	8.782	2.192	6.076
	SFT	0.995	3.431	0.023	0.065
Average	CSA	0.991	1.252	0.588	1.628
	UnCorEI	0.960	2.690	1.314	3.642
	CorEI	0.991	1.730	1.964	3.268
	MT	0.986	1.841	0.049	0.136
	PA	0.848	9.353	1.896	5.256
	SFT	0.997	2.581	0.017	0.047

CSA: Cross-sectional area (cm^2^); UnCorEI: Uncorrected echo intensity (AU); CorEI: Corrected echo intensity (AU); MT: Muscle thickness (cm); PA: Pennation angle (°); SFT: Subcutaneous adipose tissue thickness (cm); IP: Assessments taken immediately post laying down in the non-dominant lateral recumbent position; NDLR: Assessments taken 15 min after laying down in the non-dominant lateral recumbent position; SUP: Assessments taken 15 min after laying down in the supine position; DLR: Assessments taken 15 min after laying down in the dominant lateral recumbent position; ST: Assessments taken 15 min after standing up; ICC: Intraclass correlation coefficient; CV: Coefficient of variation; SEM: Standard error of measurement; MD: Minimal difference.

**Table 4:** Values for ultrasound-derived muscle morphological characteristics of the vastus lateralis after rest in different positions.

Rest Position	CSA (cm^2^)	UnCorEI (AU)	CorEI (AU)	MT (cm)	PA (°)	SFT (cm)
IP	34.91 ± 6.48	49.93 ± 6.76 *	74.58 ± 14.87 *	2.077 ± 0.445 *	17.45 ± 4.89 *	0.608 ± 0.294 *
NDLR	34.52 ± 6.58 *^,†^	49.67 ± 6.52 *	74.19 ± 15.19 *	2.083 ± 0.443 *	17.49 ± 4.48 *	0.605 ± 0.289 *
SUP	34.54 ± 6.38 *^,†^	49.09 ± 6.85 *^,†^	72.87 ± 14.69 ^†,‡^	2.071 ± 0.428 *	17.09 ± 4.41 *	0.610 ± 0.298 *
DLR	34.68 ± 6.42 *	48.52 ± 6.21 *^,†,‡^	73.14 ± 14.49 ^†,‡^	2.068 ± 0.463 *	16.71 ± 4.92 *	0.607 ± 0.293 *
ST	35.32 ± 6.72	46.67 ± 6.39	72.86 ± 15.67	2.470 ± 0.360	22.92 ± 4.57	0.646 ± 0.318

Values are presented as mean ± standard deviation. CSA: Cross-sectional area; UnCorEI: Uncorrected echo intensity; CorEI: Corrected echo intensity; MT: Muscle thickness; PA: Pennation angle; SFT: Subcutaneous adipose tissue thickness; IP: Assessments taken immediately post laying down in the non-dominant lateral recumbent position; NDLR: Assessments taken 15 min after laying down in the non-dominant lateral recumbent position; SUP: Assessments taken 15 min after laying down in the supine position; DLR: Assessments taken 15 min after laying down in the dominant lateral recumbent position; ST: Assessments taken 15 min after standing up. * Significantly different from ST (*p* < 0.05). ^†^ Significantly different from IP (*p* < 0.05). ^‡^ Significantly different from NDLR (*p* < 0.05).

**Table 5:** Associations between ultrasound-derived muscle morphological characteristics after rest in different positions and unilateral vertical jump (UVJ) outcome measures.

Morphological Variable	UVJ Variable	Best Position Predictor	*r*	*R* ^2^	SEE	*p*-Value	Other Potential Positions
CSA	Height	None	-	-	-	-	-
	PF	DLR ^†*,b*^	0.592	0.350	191.44	<0.001	IP ^†^, NDLR ^†^, SUP ^†^, ST ^†^
	Peak Power	IP ^†^	0.537	0.288	403.65	0.002	NDLR ^†^, SUP ^†^, DLR ^†^, ST ^†^
	Peak Velocity	None	-	-	-	-	-
	Total Work	IP *	0.425	0.181	94.06	0.017	NDLR *, SUP *, DLR *, ST *
	RPD	None	-	-	-	-	-
UnCorEI	Height	None	-	-	-	-	-
	PF	None	-	-	-	-	-
	Peak Power	None	-	-	-	-	-
	Peak Velocity	None	-	-	-	-	-
	Total Work	None	-	-	-	-	-
	RPD	None	-	-	-	-	-
CorEI	Height	NDLR ^†^	−0.556	0.309	2.61	0.001	IP ^†^, SUP ^†^, DLR ^†^, ST *
	PF	None	-	-	-	-	-
	Peak Power	None	-	-	-	-	-
	Peak Velocity	IP *	−0.484	0.235	0.15	0.006	NDLR *, SUP *, DLR *, ST *
	Total Work	None	-	-	-	-	-
	RPD	None	-	-	-	-	-
MT	Height	None	-	-	-	-	-
	PF	IP *	0.449	0.202	212.12	0.011	NDLR *, SUP *, DLR *, ST *
	Peak Power	ST *	0.433	0.187	431.25	0.015	IP *, NDLR *, DLR *
	Peak Velocity	None	-	-	-	-	-
	Total Work	None	-	-	-	-	-
	RPD	None	-	-	-	-	-
PA	Height	IP *^,^*^a,b^*	0.363	0.132	2.93	0.045	None
	PF	DLR *^,^*^c^*	0.453	0.205	211.67	0.010	IP *^,^*^c^*, NDLR *^,^*^c^*, SUP *^,^*^c^*
	Peak Power	IP *^,^*^c^*	0.475	0.226	420.85	0.007	NDLR *, DLR *
	Peak Velocity	IP *^,^*^b^*	0.360	0.129	0.16	0.047	None
	Total Work	None	-	-	-	-	-
	RPD	IP ^†,*c*^	0.646	0.418	2295.56	<0.001	NDLR ^†^, SUP ^†^, DLR ^†^, ST *
SFT	Height	IP ^†^	−0.565	0.319	2.60	0.001	NDLR ^†^, SUP ^†^, DLR ^†^, ST ^†^
	PF	-	-	-	-	-	-
	Peak Power	-	-	-	-	-	-
	Peak Velocity	IP *	−0.503	0.253	0.15	0.004	NDLR *, SUP *, DLR *, ST *
	Total Work	-	-	-	-	-	-
	RPD	-	-	-	-	-	-

Associations between ultrasound-derived morphological characteristics and UVJ outcome measures based on the rest position having the greatest shared variance with the outcome variable. The morphological variable is presented first, followed by the rest position that best predicts the dependent variable. “Other Potential Positions” denotes rest positions also having a significant association with the dependent variable. “None” indicates that the specific measure of morphology was not a significant predictor of jump performance after rest in any position. *r:* Pearson’s correlation coefficient; *R*^2^: Shared variance; SEE: Standard error of the estimate; CSA: Cross-sectional area; UnCorEI: Uncorrected echo intensity; CorEI: Corrected echo intensity; MT: Muscle thickness; PA: Pennation angle; SFT: Subcutaneous adipose tissue thickness; PF: Peak force; RPD: Rate of power development; IP: Assessments taken immediately post laying down in the non-dominant lateral recumbent position; NDLR: Assessments taken 15 min after laying down in the non-dominant lateral recumbent position; SUP: Assessments taken 15 min after laying down in the supine position; DLR: Assessments taken 15 min after laying down in the dominant lateral recumbent position; ST: Assessments taken 15 min after standing up. Statistically significant (*p* ≤ 0.05) correlation magnitudes were quantified using the following descriptors [44]: * Moderate. ^†^ Large. Differences between correlation coefficients were examined using the Williams modification of the Hotelling test [45]: *^a^* Signficantly stronger than DLR (*p* ≤ 0.05); *^b^* Signficantly stronger than SUP (*p* ≤ 0.05); *^c^* Signficantly stronger than ST (*p* ≤ 0.05).

**Table 6:** Associations between muscle ultrasound-derived muscle morphological characteristics after rest in different positions and isokinetic variables after correcting for Biodex dynamometer arm length.

Morphological Variable	Isokinetic Variable	Best Position Predictor	*r*	*R* ^2^	SEE	*p*-Value	Other Potential Positions
CSA	IsokPF (60°·s^−1^)	None	-	-	-	-	-
	IsokPF (180°·s^−1^)	None	-	-	-	-	-
	IsokPF (240°·s^−1^)	None	-	-	-	-	-
UnCorEI	IsokPF (60°·s^−1^)	None	-	-	-	-	-
	IsokPF (180°·s^−1^)	None	-	-	-	-	-
	IsokPF (240°·s^−1^)	None	-	-	-	-	-
CorEI	IsokPF (60°·s^−1^)	None	-	-	-	-	-
	IsokPF (180°·s^−1^)	None	-	-	-	-	-
	IsokPF (240°·s^−1^)	None	-	-	-	-	-
MT	IsokPF (60°·s^−1^)	None	-	-	-	-	-
	IsokPF (180°·s^−1^)	DLR *	0.457	0.209	84.88	0.010	IP *, NDLR *, SUP *, ST *
	IsokPF (240°·s^−1^)	DLR *	0.398	0.158	72.32	0.027	IP *, NDLR *
PA	IsokPF (60°·s^−1^)	DLR *^,^*^a^*	0.358	0.129	139.81	0.048	None
	IsokPF (180°·s^−1^)	ST *	0.447	0.200	85.35	0.012	IP *, NDLR *, DLR *, SUP *
	IsokPF (240°·s^−1^)	DLR *	0.379	0.144	72.95	0.035	None
SFT	IsokPF (60°·s^−1^)	None	-	-	-	-	-
	IsokPF (180°·s^−1^)	None	-	-	-	-	-
	IsokPF (240°·s^−1^)	None	-	-	-	-	-

Associations between ultrasound-derived morphological characteristics and UVJ outcome measures based on the rest position having the greatest shared variance with the outcome variable. The morphological variable is presented first, followed by the rest position that best predicts the dependent variable. “Other Potential Positions” denotes rest positions also having a significant association with the dependent variable. “None” indicates that the specific measure of morphology was not a significant predictor of jump performance after rest in any position. *r*: Pearson’s correlation coefficient; *R*^2^: Shared variance; SEE: Standard error of the estimate; CSA: Cross-sectional area; UnCorEI: Uncorrected echo intensity; CorEI: Corrected echo intensity; MT: Muscle thickness; PA: Pennation angle; SFT: Subcutaneous adipose tissue thickness; PF: Peak force; RPD: Rate of power development; IP: Assessments taken immediately post laying down in the non-dominant lateral recumbent position; NDLR: Assessments taken 15 min after laying down in the non-dominant lateral recumbent position; SUP: Assessments taken 15 min after laying down in the supine position; DLR: Assessments taken 15 min after laying down in the dominant lateral recumbent position; ST: Assessments taken 15 min after standing up. Statistically significant (*p* ≤ 0.05) correlation magnitudes were quantified using the following descriptors [44]: * Moderate. Differences between correlation coefficients were examined using the Williams modification of the Hotelling test [45]: *^a^* Signficantly stronger than SUP (*p* ≤ 0.05).

**Table 7:** Associations between ultrasound-derived muscle morphological characteristics after rest in different positions and unilateral strength.

Morphological Variable	Strength Variable	Best Position Predictor	*r*	*R* ^2^	SEE	*p*-Value	Other Potential Positions
CSA	1-RM Leg Press	NDLR ^‡^	0.713	0.508	80.54	<0.001	IP ^‡^, SUP ^†^, DLR ^†^, ST ^†^
UnCorEI		None	-	-	-	-	-
CorEI		None	-	-	-	-	-
MT		IP ^‡^	0.722	0.522	79.42	<0.001	NDLR ^†^ SUP ^†^, DLR ^†^, ST ^†^
PA		NDLR *	0.427	0.182	103.83	0.017	IP *, SUP *, DLR *
SFT		None	-	-	-	-	-

Associations between ultrasound-derived morphological characteristics and maximal unilateral strength based on the rest position having the greatest shared variance with the dependent variable. The morphological variable is presented first, followed by the rest position that best predicts the dependent variable. “None” indicates that the specific measure of morphology was not a significant predictor of maximal strength after rest in any position. “Other Potential Positions” denotes rest positions also having a significant association with the dependent variable. *r*: Pearson’s correlation coefficient; *R*^2^: Shared variance; SEE: Standard error of the estimate; CSA: Cross-sectional area; UnCorEI: Uncorrected echo intensity; CorEI: Corrected echo intensity; MT: Muscle thickness; PA: Pennation angle; SFT: Subcutaneous adipose tissue thickness; IP: Assessments taken immediately post laying down in the non-dominant lateral recumbent position; NDLR: Assessments taken 15 min after laying down in the non-dominant lateral recumbent position; SUP: Assessments taken 15 min after laying down in the supine position; DLR: Assessments taken 15 min after laying down in the dominant lateral recumbent position; ST: Assessments taken 15 min after standing up. Statistically significant (*p* ≤ 0.05) correlation magnitudes were quantified using the following descriptors [44]: *Moderate. ^†^ Large. ^‡^ Very Large.
